# Supplementary material for: Human DUX4 and mouse Dux interact with STAT1 and broadly inhibit interferon-stimulated gene induction
Source: eLife. 2023 Apr 24;12:e82057. doi: 10.7554/eLife.82057 (PMC10195082; doi:10.7554/eLife.82057)
Supplement: Figure 3—source data 8. — Western blot showing anti-PPP2R1A signal. * marks correct size band. Blot was physically cut to probe with multiple antibodies, multiple separate blots were imaged in this exposure/file. Bottom right blot (boxed in green) is relevant for this figure and was probed with PPP2R1A. Signal from ECL only appears in the chemiluminescence channel. Protein ladder appears in white light channel. [file elife-82057-fig3-data8.zip › Figure3-SourceData8.pdf]

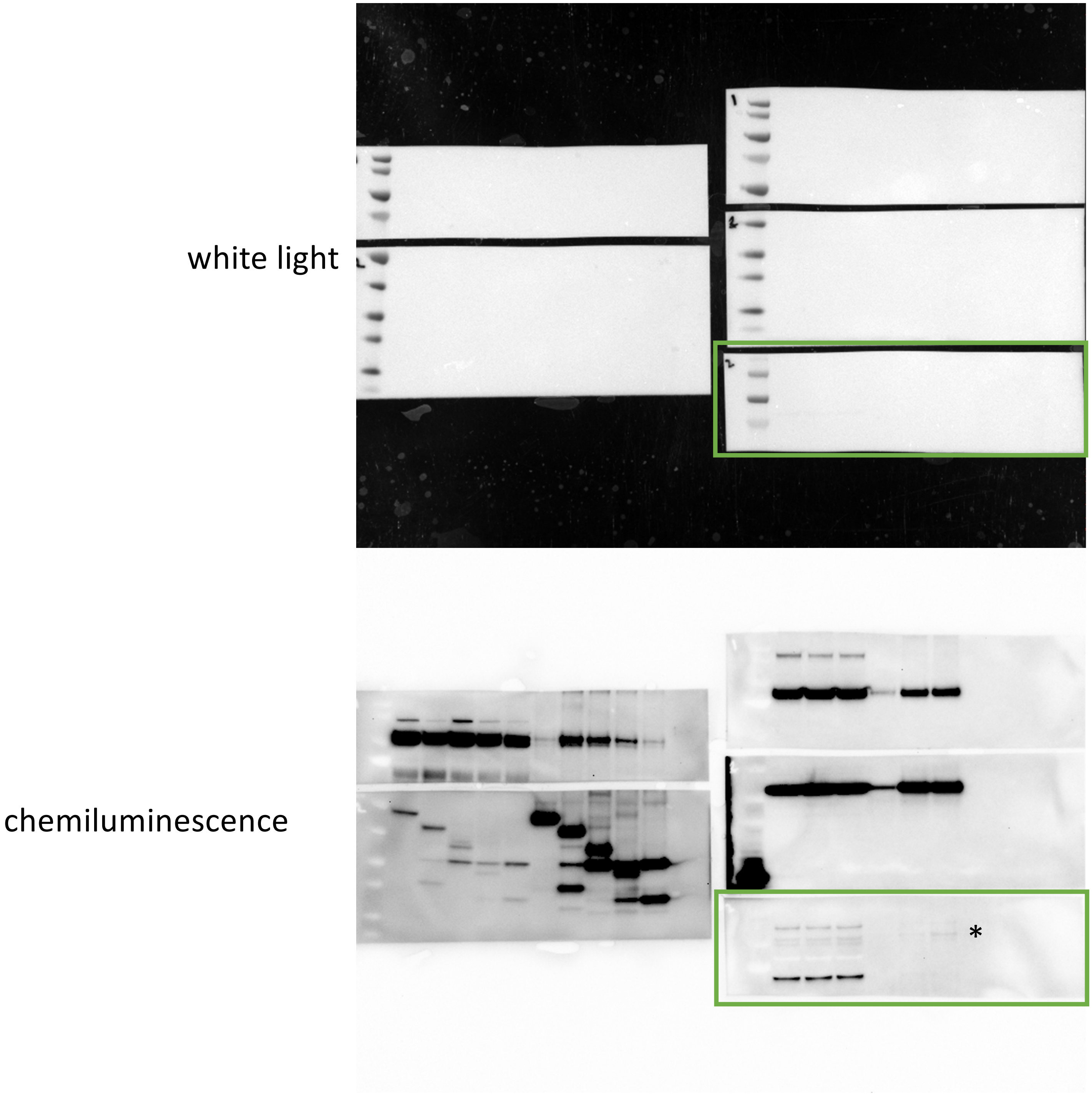

Figure 3 Source Data 8. Validation co-IP from inducible MB135 cell lines, anti-PPP2R1A. Western blot showing anti-PPP2R1A signal. * marks correct size band. Blot was physically cut to probe with multiple antibodies, multiple separate blots were imaged in this exposure/file. BOTTOM RIGHT BLOT (boxed in green) is relevant for this figure and was probed with PPP2R1A. Signal from ECL only appears in the chemiluminescence channel. Protein ladder appears in white light channel.
