# Supplementary material for: Human DUX4 and mouse Dux interact with STAT1 and broadly inhibit interferon-stimulated gene induction
Source: eLife. 2023 Apr 24;12:e82057. doi: 10.7554/eLife.82057 (PMC10195082; doi:10.7554/eLife.82057)
Supplement: Figure 3—source data 13. — Western blot showing anti-TRIM28 signal. * marks correct size band. Blot was physically cut to probe with multiple antibodies, multiple separate blots were imaged in this exposure/file. Top blot (boxed in green) is relevant for this figure and was probed with anti-TRIM28. Signal from ECL only appears in the chemiluminescence channel. Protein ladder appears in white light channel. [file elife-82057-fig3-data13.zip › Figure3-SourceData13.pdf]

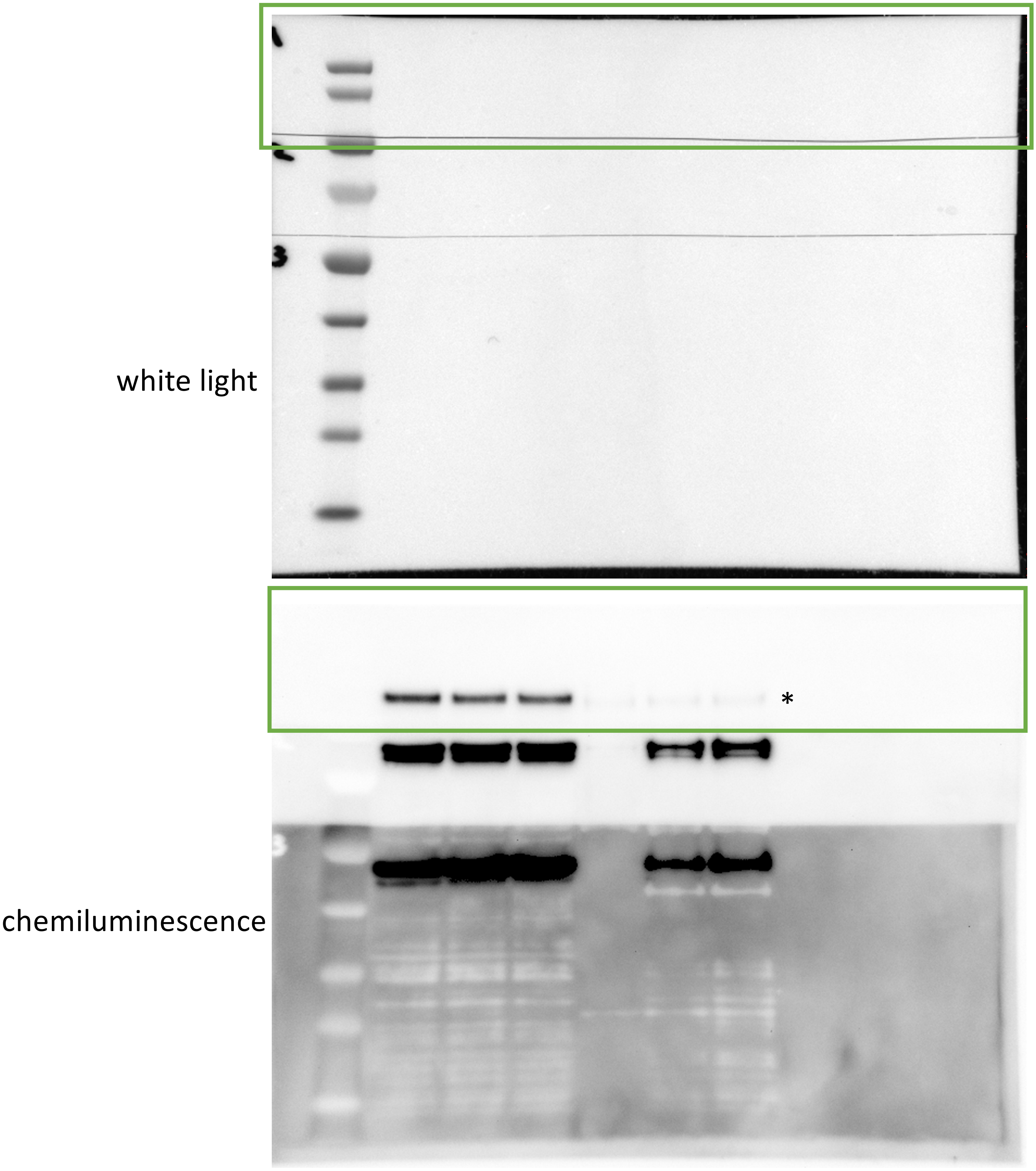

Figure 3 Source Data 13. Validation co-IP from inducible MB135 cell lines, anti-TRIM28. Western blot showing anti-TRIM28 signal. * marks correct size band. Blot was physically cut to probe with multiple antibodies, multiple separate blots were imaged in this exposure/file. TOP BLOT (boxed in green) is relevant for this figure and was probed with anti-TRIM28. Signal from ECL only appears in the chemiluminescence channel. Protein ladder appears in white light channel.
