# Supplementary material for: Human DUX4 and mouse Dux interact with STAT1 and broadly inhibit interferon-stimulated gene induction
Source: eLife. 2023 Apr 24;12:e82057. doi: 10.7554/eLife.82057 (PMC10195082; doi:10.7554/eLife.82057)
Supplement: Figure 4—source data 1. — Western blot showing anti-STAT1 signal for Figure 4A. * marks correct size band. Blot was physically cut to probe with multiple antibodies, multiple separate blots were imaged in this exposure/file. Top left blot (boxed in green) is relevant for this figure and was probed with anti-STAT1. The multiple bands represent the alpha (upper) and beta (lower) isoforms of STAT1. [file elife-82057-fig4-data1.zip › Figure4-SourceData1.pdf]

chemiluminescence

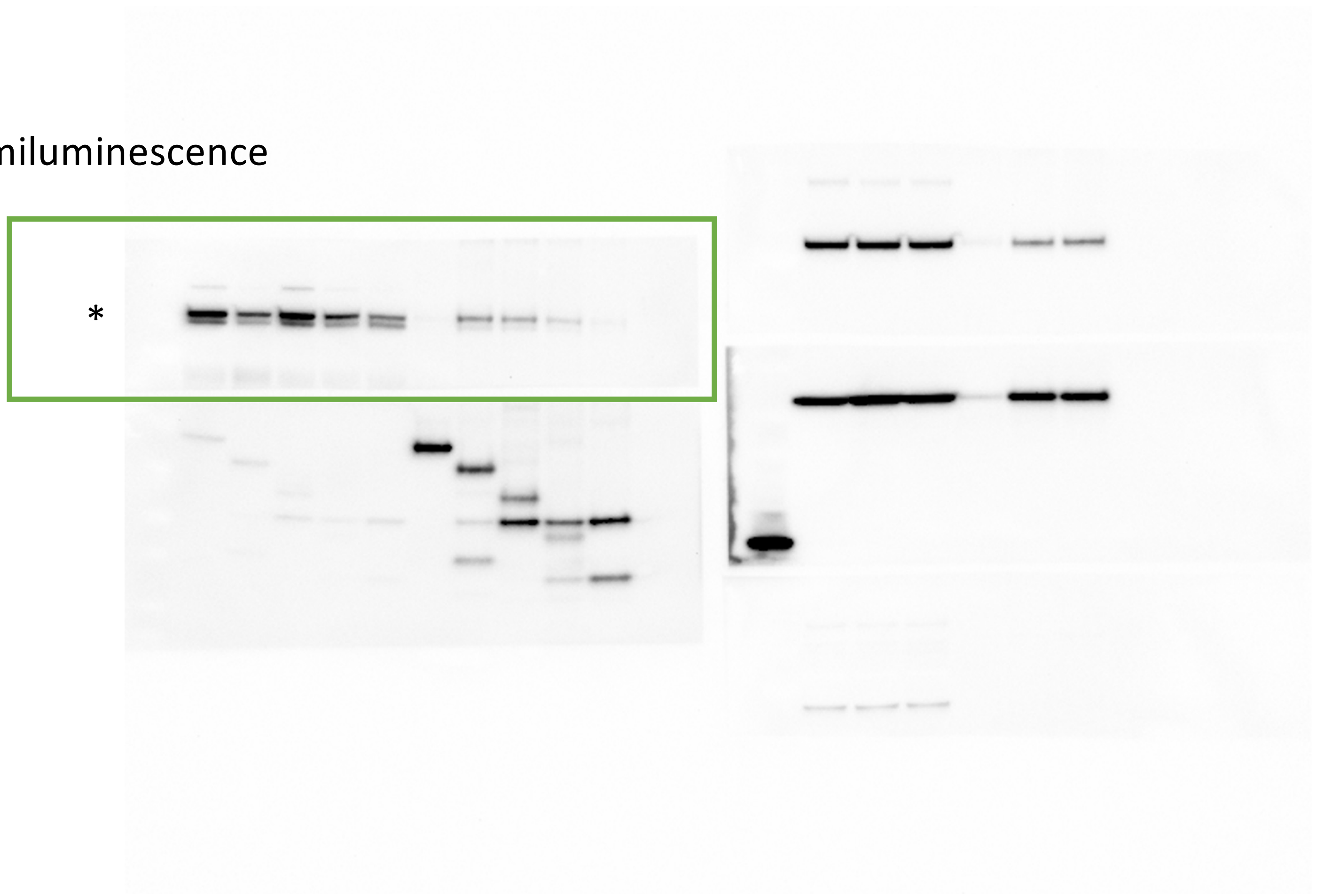

Figure 4 Source Data 1. Co-IP from inducible MB135 cell lines, anti-STAT1. Western blot showing anti-STAT1 signal for Figure 4a. * marks correct size band. Blot was physically cut to probe with multiple antibodies, multiple separate blots were imaged in this exposure/file. TOP LEFT BLOT (boxed in green) is relevant for this figure and was probed with anti-STAT1. The multiple bands represent the alpha (upper) and beta (lower) isoforms of STAT1.
