# Supplementary material for: Human DUX4 and mouse Dux interact with STAT1 and broadly inhibit interferon-stimulated gene induction
Source: eLife. 2023 Apr 24;12:e82057. doi: 10.7554/eLife.82057 (PMC10195082; doi:10.7554/eLife.82057)
Supplement: Figure 4—source data 2. — Western blot showing anti-pSTAT1(Y701) signal for Figure 4A. * marks correct size band. Blot was physically cut to probe with multiple antibodies, multiple unrelated blots were imaged in this exposure/file. Top blot (boxed in green) is relevant for this figure and was probed with anti-pSTAT1(Y701). Protein ladder appears in white light channel. Signal from ECL only appears in the chemiluminescence channel. The multiple bands represent the alpha (upper) and beta (lower) isoforms of STAT1. [file elife-82057-fig4-data2.zip › Figure4-SourceData2.pdf]

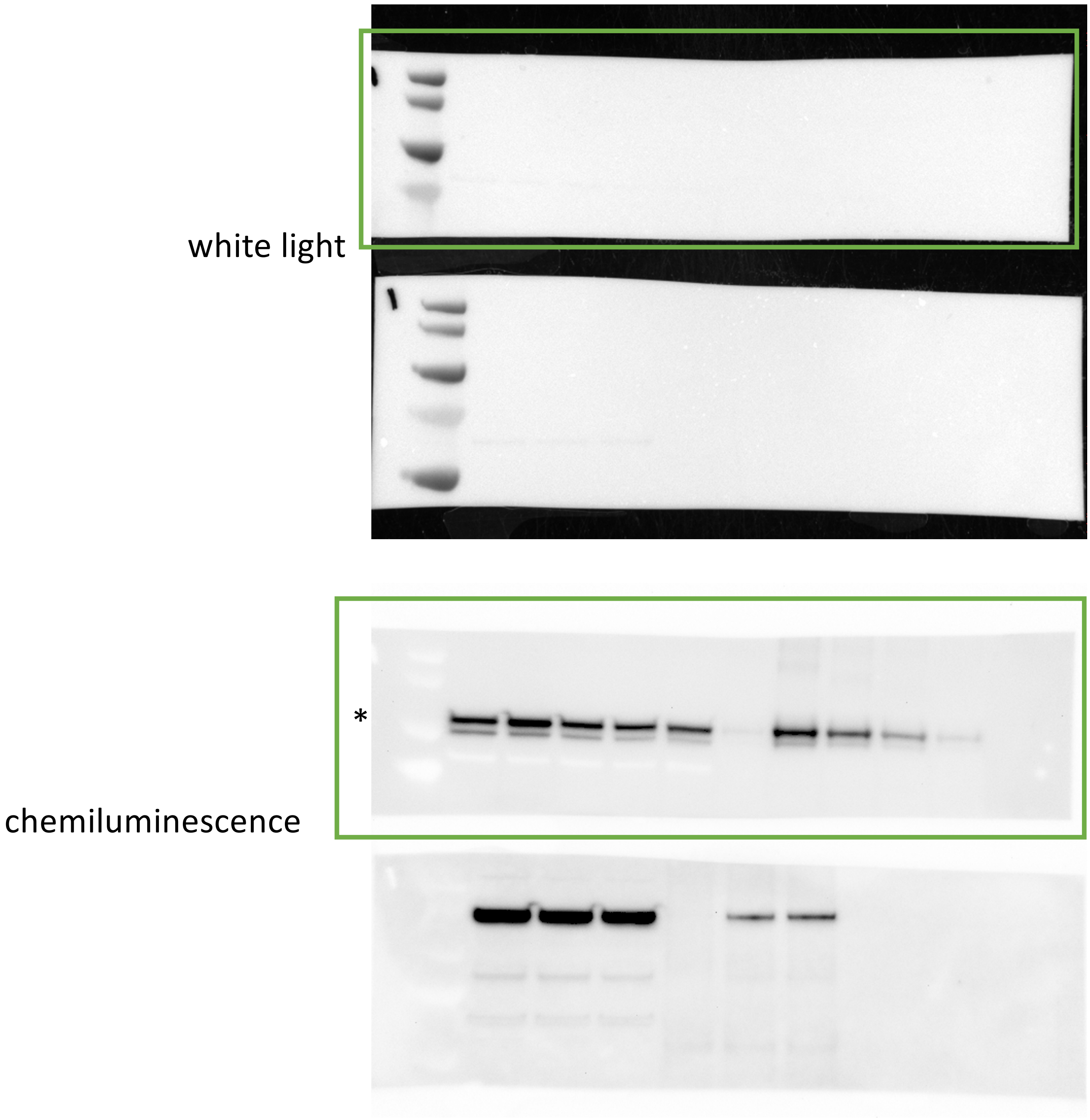

Figure 4 Source Data 2. Co-IP from inducible MB135 cell lines, anti-pSTAT1(Y701). Western blot showing anti-pSTAT1(Y701) signal for Figure 4a. * marks correct size band. Blot was physically cut to probe with multiple antibodies, multiple unrelated blots were imaged in this exposure/file. TOP BLOT (boxed in green) is relevant for this figure and was probed with anti-pSTAT1(Y701). Protein ladder appears in white light channel. Signal from ECL only appears in the chemiluminescence channel. The multiple bands represent the alpha (upper) and beta (lower) isoforms of STAT1.
