# Supplementary material for: Human DUX4 and mouse Dux interact with STAT1 and broadly inhibit interferon-stimulated gene induction
Source: eLife. 2023 Apr 24;12:e82057. doi: 10.7554/eLife.82057 (PMC10195082; doi:10.7554/eLife.82057)
Supplement: Figure 4—source data 3. — Western blot showing anti-pSTAT1(S727) signal for Figure 4A. * marks correct size band. This blot is probed with anti-pSTAT1(S727). Protein ladder appears in white light channel. Signal from ECL only appears in the chemiluminescence channel. [file elife-82057-fig4-data3.zip › Figure4-SourceData3.pdf]

white light

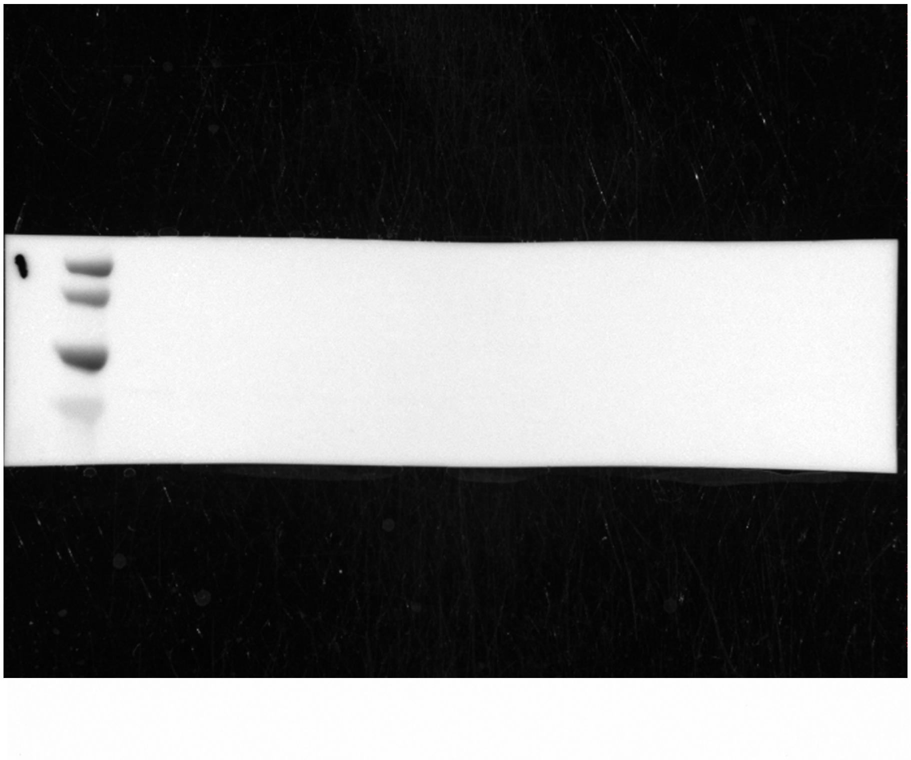

chemiluminescence

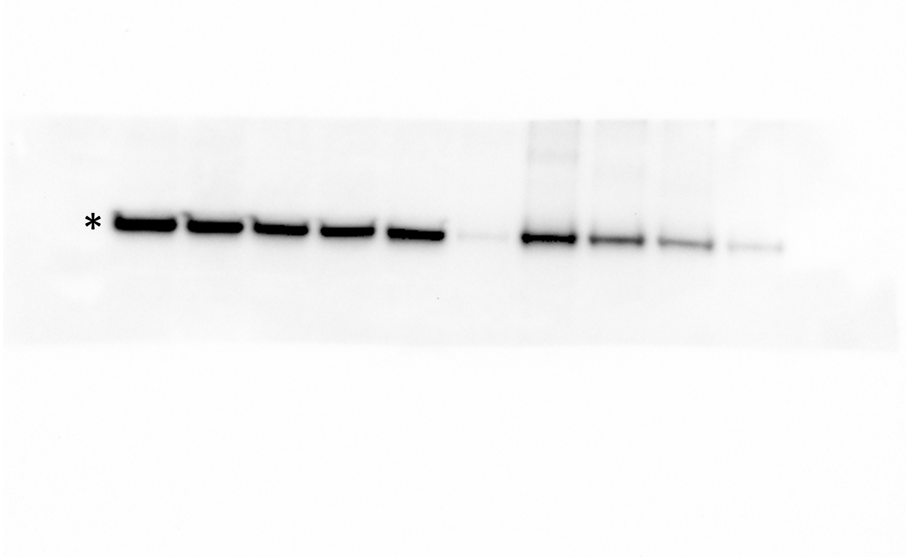

Figure 4 Source Data 3. Co-IP from inducible MB135 cell lines, anti-pSTAT1(S727). Western blot showing anti-pSTAT1(S727) signal for Figure 4a. * marks correct size band. This blot is probed with anti-pSTAT1(S727). Protein ladder appears in white light channel. Signal from ECL only appears in the chemiluminescence channel.
