# Supplementary material for: Human DUX4 and mouse Dux interact with STAT1 and broadly inhibit interferon-stimulated gene induction
Source: eLife. 2023 Apr 24;12:e82057. doi: 10.7554/eLife.82057 (PMC10195082; doi:10.7554/eLife.82057)
Supplement: Figure 4—source data 4. — Western blot showing anti-FLAG signal for Figure 4A. * marks correct size band. Blot was physically cut to probe with multiple antibodies, multiple unrelated blots were imaged in this exposure/file. Bottom left blot (boxed in green) is probed with anti-FLAG. Protein ladder appears in white light channel. Signal from ECL only appears in the chemiluminescence channel. [file elife-82057-fig4-data4.zip › Figure4-SourceData4.pdf]

white light

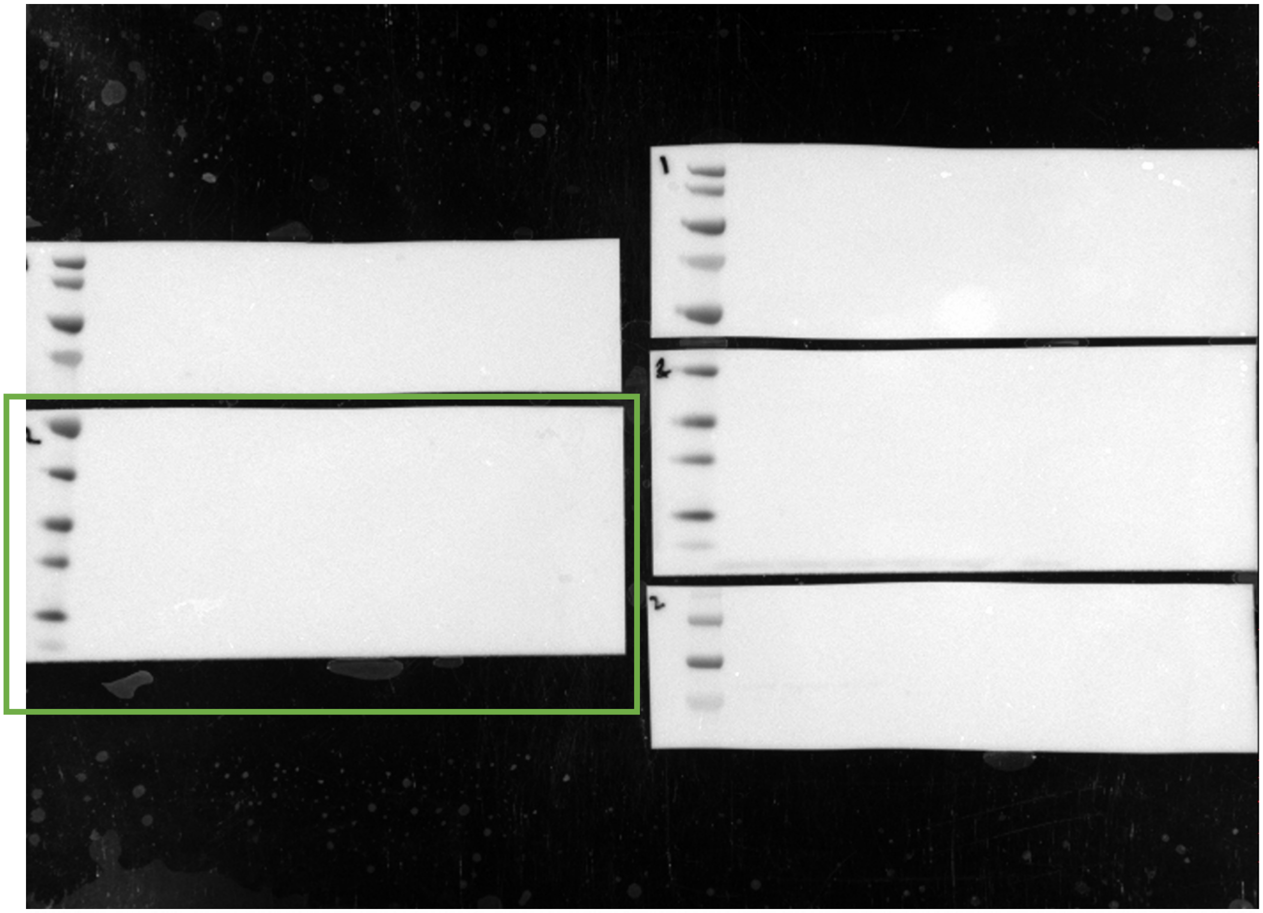

chemiluminescence

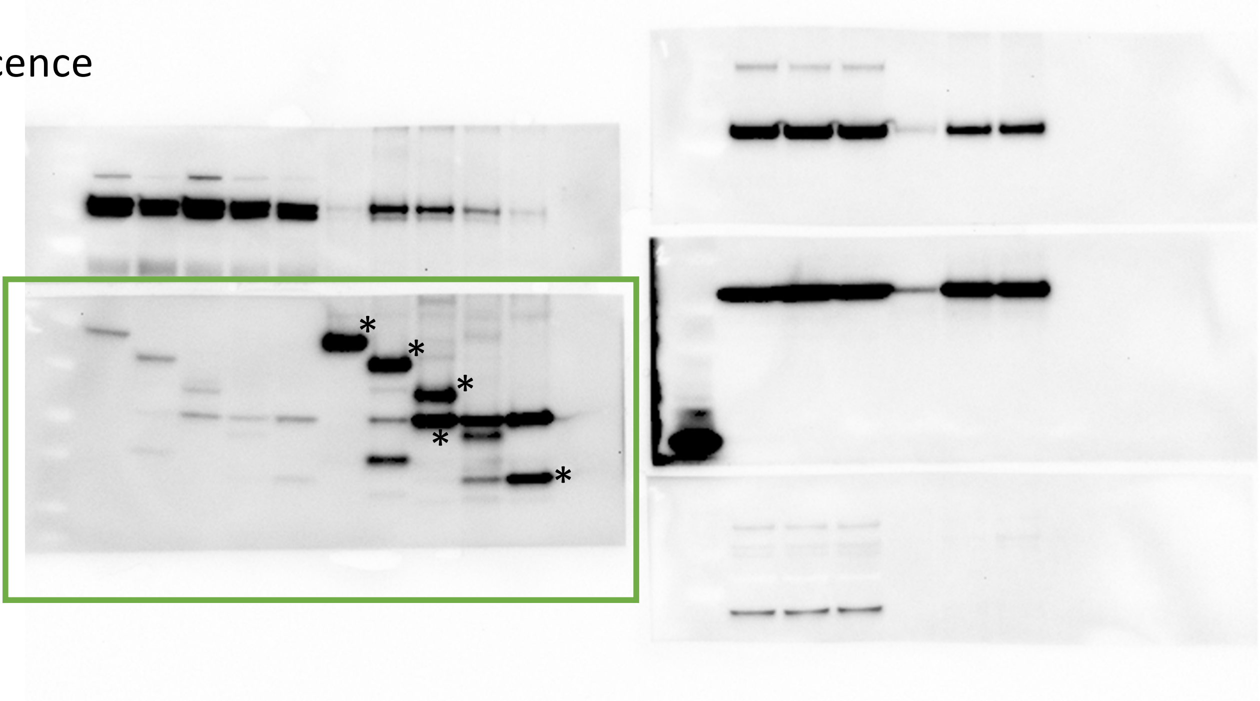

Figure 4 Source Data 4. Co-IP from inducible MB135 cell lines, anti-FLAG. Western blot showing anti-FLAG signal for Figure 4a. * marks correct size band. Blot was physically cut to probe with multiple antibodies, multiple unrelated blots were imaged in this exposure/file. BOTTOM LEFT BLOT (boxed in green) is probed with anti-FLAG. Protein ladder appears in white light channel. Signal from ECL only appears in the chemiluminescence channel.
