# Supplementary material for: Human DUX4 and mouse Dux interact with STAT1 and broadly inhibit interferon-stimulated gene induction
Source: eLife. 2023 Apr 24;12:e82057. doi: 10.7554/eLife.82057 (PMC10195082; doi:10.7554/eLife.82057)
Supplement: Figure 4—source data 6. — Western blot showing anti-STAT1 signal for Figure 4B. * marks correct size band. Blot was physically cut to probe with multiple antibodies, multiple unrelated blots were imaged in this exposure/file. Middle blot (boxed in green) is probed with anti-STAT1 to detect the INDUCIBLE MYC-tagged STAT1 transgene. Protein ladder only appears in the ‘white light’ exposure. Signal from ECL only appears in the chemiluminescence channel. [file elife-82057-fig4-data6.zip › Figure4-SourceData6.pdf]

white light

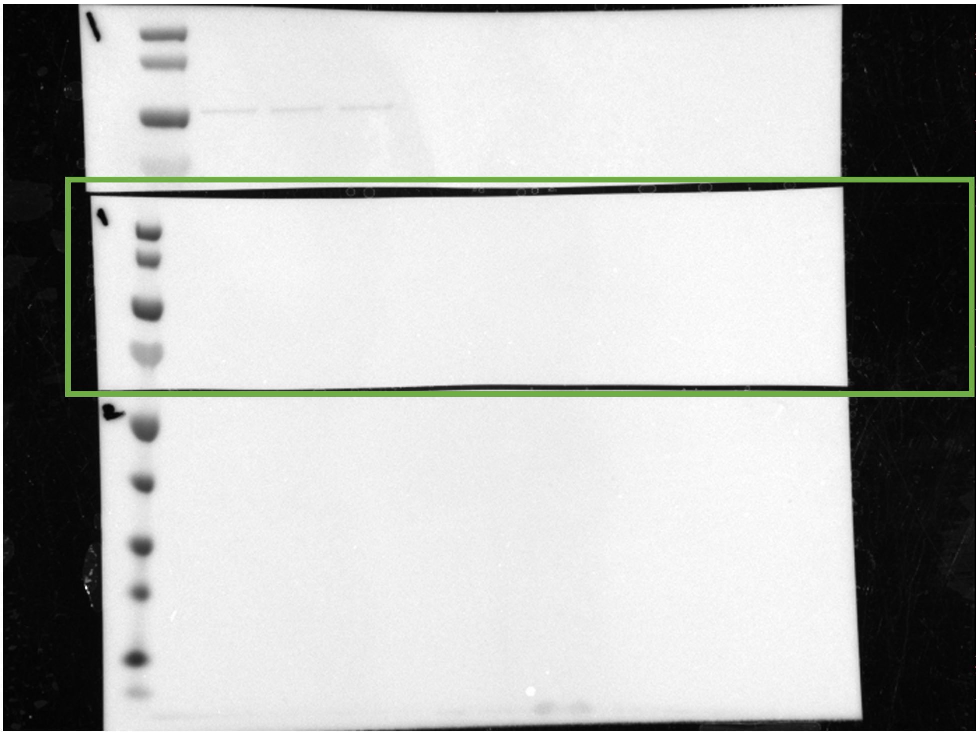

chemiluminescence

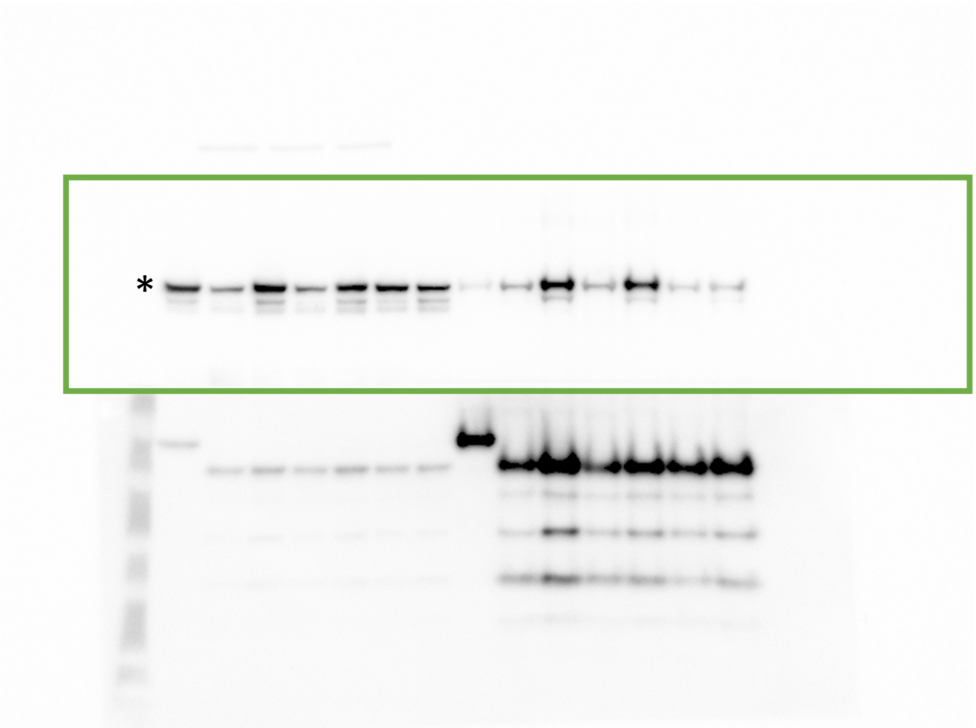

Figure 4 Source Data 6. Co-IP from dual-inducible MB135 cell lines, anti-STAT1. Western blot showing anti-STAT1 signal for Figure 4b. * marks correct size band. Blot was physically cut to probe with multiple antibodies, multiple unrelated blots were imaged in this exposure/file. MIDDLE BLOT (boxed in green) is probed with anti-STAT1 to detect the INDUCIBLE MYC-tagged STAT1 transgene. Protein ladder only appears in the “white light” exposure, signal from ECL only appears in the chemiluminescence channel.
