# Supplementary material for: Human DUX4 and mouse Dux interact with STAT1 and broadly inhibit interferon-stimulated gene induction
Source: eLife. 2023 Apr 24;12:e82057. doi: 10.7554/eLife.82057 (PMC10195082; doi:10.7554/eLife.82057)
Supplement: Figure 4—source data 7. — Western blot showing anti-pSTAT1(Y701) signal for Figure 4B. * marks correct size band. Blot is probed with anti-pSTAT1(Y701) to detect the phosphorylated INDUCIBLE MYC-tagged STAT1 or MYC-tagged STAT1-mutant transgene. Protein ladder only appears in the ‘white light’ exposure. Signal from ECL only appears in the chemiluminescence channel. [file elife-82057-fig4-data7.zip › Figure4-SourceData7.pdf]

white light

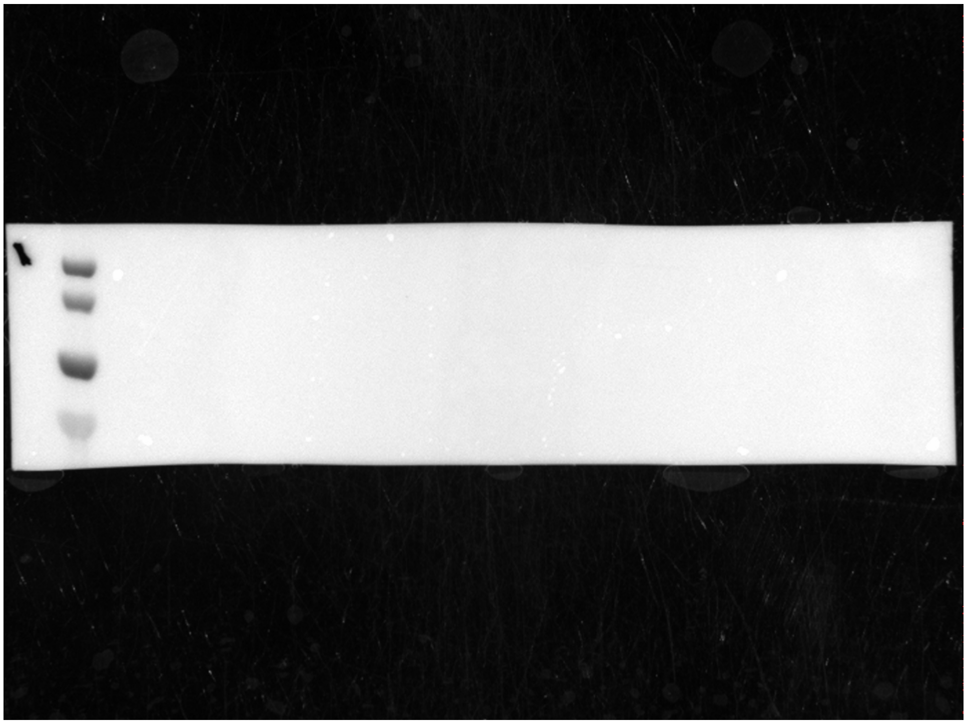

chemiluminescence

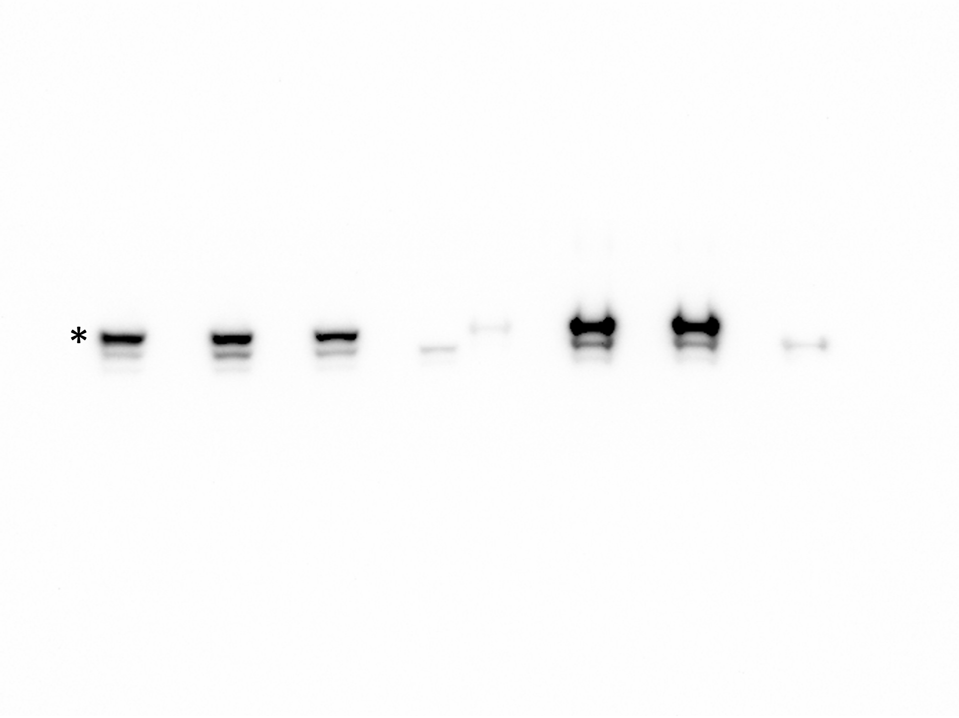

Figure 4 Source Data 7. Co-IP from dual-inducible MB135 cell lines, anti-pSTAT1(Y701). Western blot showing anti-pSTAT1(Y701) signal for Figure 4b. * marks correct size band. Blot is probed with anti-pSTAT1(Y701) to detect the phosphorylated INDUCIBLE MYC-tagged STAT1 or MYC-tagged STAT1-mutant transgene. Protein ladder only appears in the “white light” exposure, signal from ECL only appears in the chemiluminescence channel.
