# Supplementary material for: Human DUX4 and mouse Dux interact with STAT1 and broadly inhibit interferon-stimulated gene induction
Source: eLife. 2023 Apr 24;12:e82057. doi: 10.7554/eLife.82057 (PMC10195082; doi:10.7554/eLife.82057)
Supplement: Figure 4—source data 9. — Western blot showing anti-FLAG signal for Figure 4B. * marks correct size band. Blot was physically cut to probe with multiple antibodies, multiple unrelated blots were imaged in this exposure/file. Lower blot (boxed in green) is probed with anti-FLAG to detect the INDUCIBLE FLAG-tagged DUXB or DUX4-CTD transgene. Protein ladder only appears in the ‘white light’ exposure. Signal from ECL only appears in the chemiluminescence channel. [file elife-82057-fig4-data9.zip › Figure4-SourceData9.pdf]

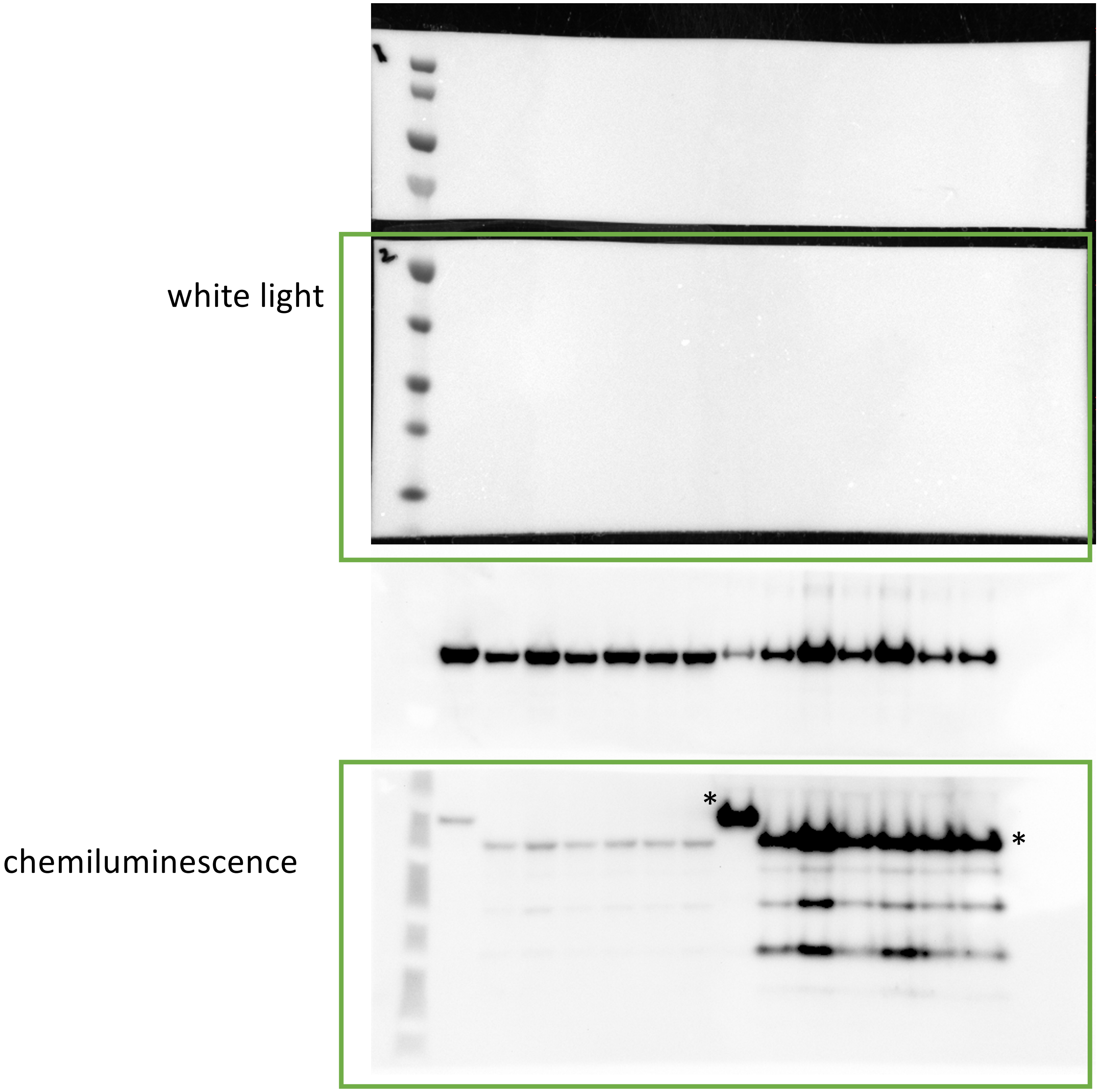

Figure 4 Source Data 9. Co-IP from dual-inducible MB135 cell lines, anti-FLAG. Western blot showing anti-FLAG signal for Figure 4b. * marks correct size band. Blot was physically cut to probe with multiple antibodies, multiple unrelated blots were imaged in this exposure/file. LOWER BLOT (boxed in green) is probed with anti-FLAG to detect the INDUCIBLE FLAG-tagged DUXB or DUX4-CTD transgene. Protein ladder only appears in the "white light" exposure, signal from ECL only appears in the chemiluminescence channel.
