# Supplementary material for: Human DUX4 and mouse Dux interact with STAT1 and broadly inhibit interferon-stimulated gene induction
Source: eLife. 2023 Apr 24;12:e82057. doi: 10.7554/eLife.82057 (PMC10195082; doi:10.7554/eLife.82057)
Supplement: Figure 6—source data 1. — Western blot showing anti-CIC signal for Figure 6B. * marks correct size band. Blot was probed with anti-CIC. NOTE: gel was loaded and transferred with samples ordered as labeled here. The image has been flipped in the article, and the labels flipped appropriately to mirror the protein layout in the Kitra-SRS experiment. Protein ladder only appears in the ‘white light’ exposure. Signal from ECL only appears in the chemiluminescence channel. [file elife-82057-fig6-data1.zip › Figure6-SourceData1.pdf]

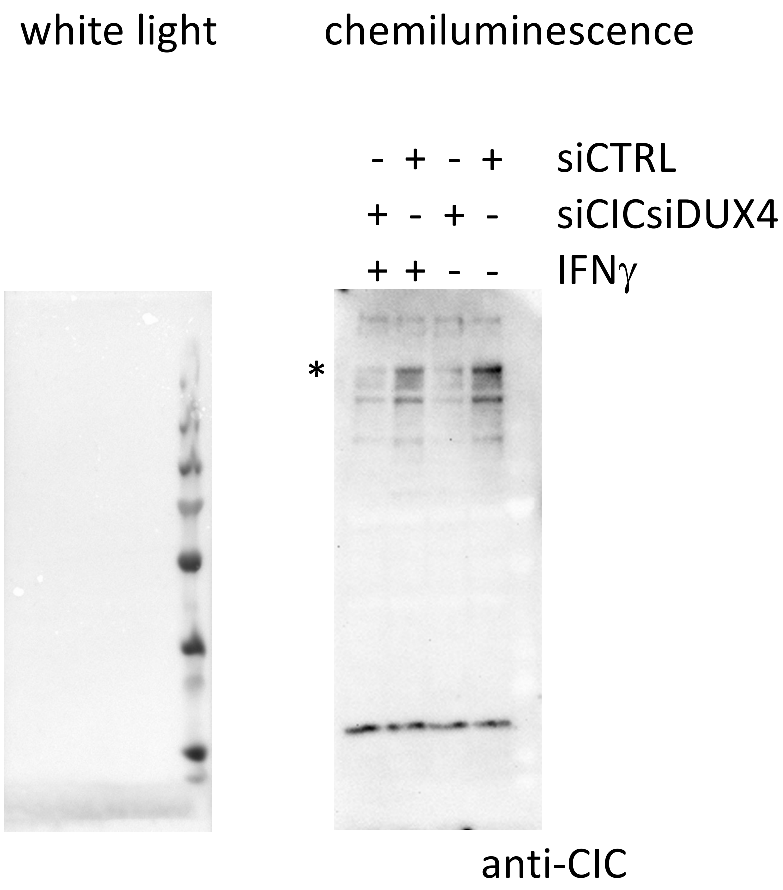

Figure 6 Source Data 1. Parental MB135 anti-CIC. Western blot showing anti-CIC signal for Figure 6b. * marks correct size band. Blot was probed with anti-CIC. NOTE: gel was loaded and transferred with samples ordered as labelled here. The image has been flipped in the manuscript and the labels flipped appropriately to mirror the protein layout in the Kitra-SRS experiment. Protein ladder only appears in the "white light" exposure, signal from ECL only appears in the chemiluminescence channel.
