# Supplementary material for: Human DUX4 and mouse Dux interact with STAT1 and broadly inhibit interferon-stimulated gene induction
Source: eLife. 2023 Apr 24;12:e82057. doi: 10.7554/eLife.82057 (PMC10195082; doi:10.7554/eLife.82057)
Supplement: Figure 6—source data 4. — Western blot showing anti-GAPDH signal for Figure 6B. * marks band of correct size. Blot was cut into two pieces, this piece was probed with anti-GAPDH. Protein ladder only appears in the ‘white light’ exposure. Signal from ECL only appears in the chemiluminescence channel. [file elife-82057-fig6-data4.zip › Figure6-SourceData4.pdf]

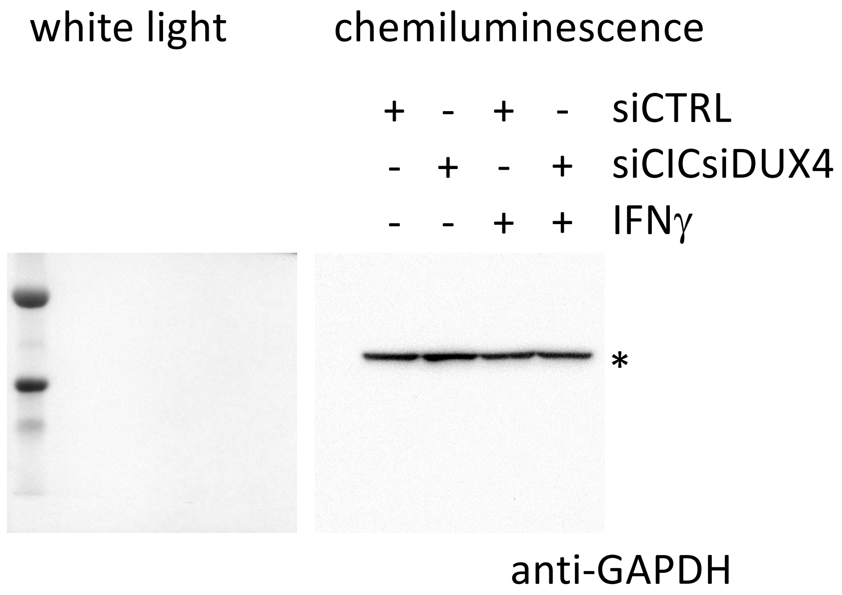

Figure 6 Source Data 4. KitraSRS anti-GAPDH. Western blot showing anti-GAPDH signal for Figure 6b. "*" marks band of correct size. Blot was cut into two pieces, this piece was probed with anti-GAPDH. Protein ladder only appears in the "white light" exposure, signal from ECL only appears in the chemiluminescence channel.
