# Supplementary material for: Human DUX4 and mouse Dux interact with STAT1 and broadly inhibit interferon-stimulated gene induction
Source: eLife. 2023 Apr 24;12:e82057. doi: 10.7554/eLife.82057 (PMC10195082; doi:10.7554/eLife.82057)
Supplement: Figure 7—source data 1. — Western blot showing anti-STAT1 signal for Figure 7B. * marks correct size bands. Blot was probed intact for STAT1. Protein ladder only appears in the ‘white light’ exposure. Signal from ECL only appears in the chemiluminescence channel. The double bands marked by the * represent the alpha (upper) and beta (lower) isoforms of endogenous STAT1. [file elife-82057-fig7-data1.zip › Figure7-SourceData1.pdf]

white light:

chemiluminescence:

*

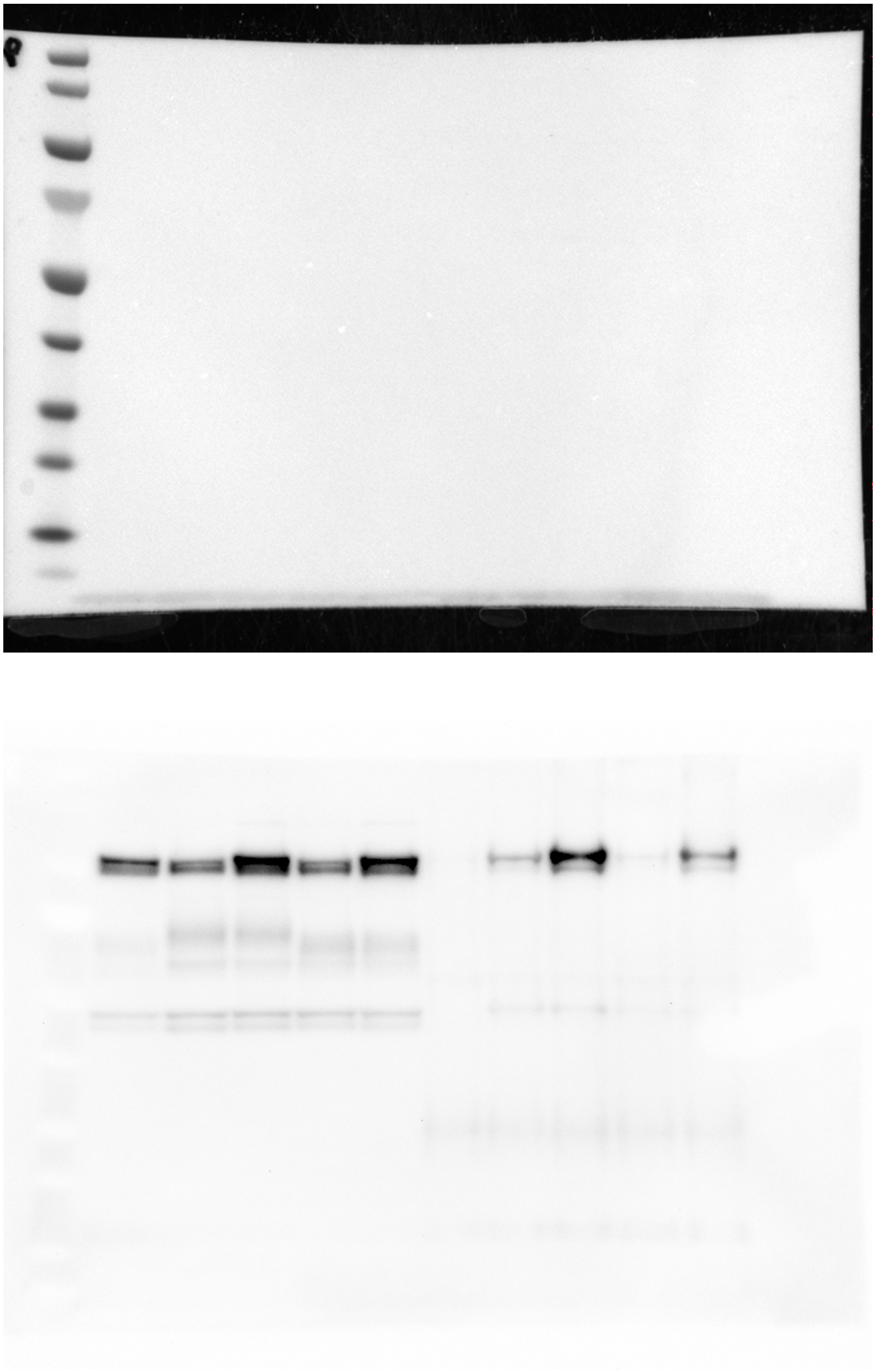

Figure 7 Source Data 1. Mouse Dux co-IP, anti-STAT1. Western blot showing anti-STAT1 signal for Figure 7b. * marks correct size bands. Blot was probed intact for STAT1. Protein ladder only appears in the “white light” exposure, signal from ECL only appears in the chemiluminescence channel. The double-bands marked by the * represent the alpha (upper) and beta (lower) isoforms of endogenous STAT1.
