# Supplementary material for: Human DUX4 and mouse Dux interact with STAT1 and broadly inhibit interferon-stimulated gene induction
Source: eLife. 2023 Apr 24;12:e82057. doi: 10.7554/eLife.82057 (PMC10195082; doi:10.7554/eLife.82057)
Supplement: Figure 7—source data 2. — Western blot showing anti-pSTAT1(Y701) signal for Figure 7B. * marks correct size bands. Blot was stripped from previous exposure and re-probed with anti-pSTAT1(Y701). Protein ladder only appears in the ‘white light’ exposure. Signal from ECL only appears in the chemiluminescence channel. The double bands marked by the * represent the alpha (upper) and beta (lower) isoforms of endogenous STAT1. [file elife-82057-fig7-data2.zip › Figure7-SourceData2.pdf]

white light:

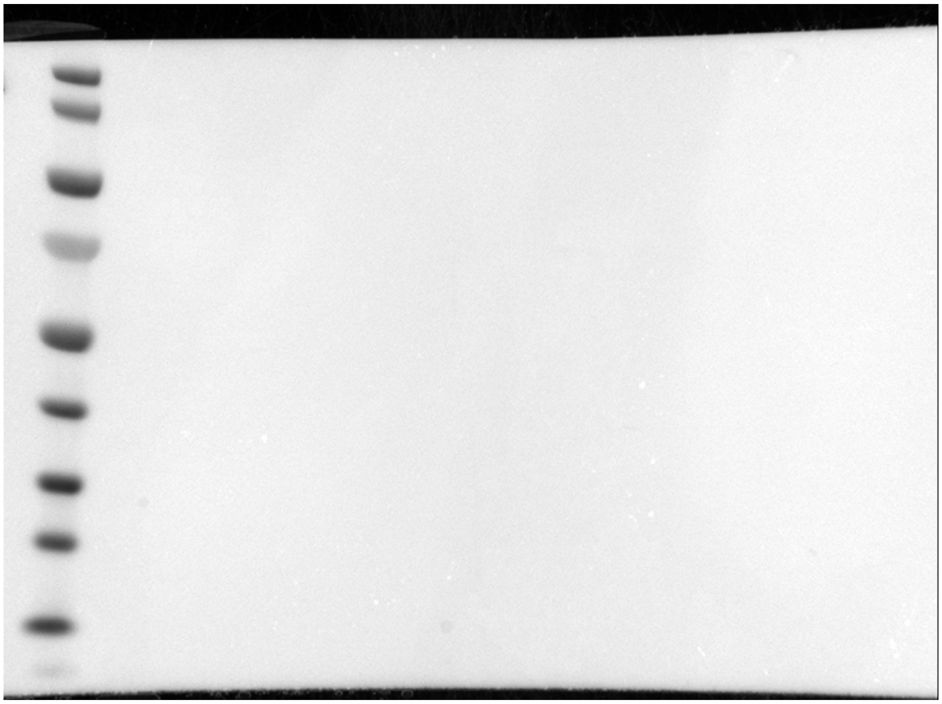

chemiluminescence:

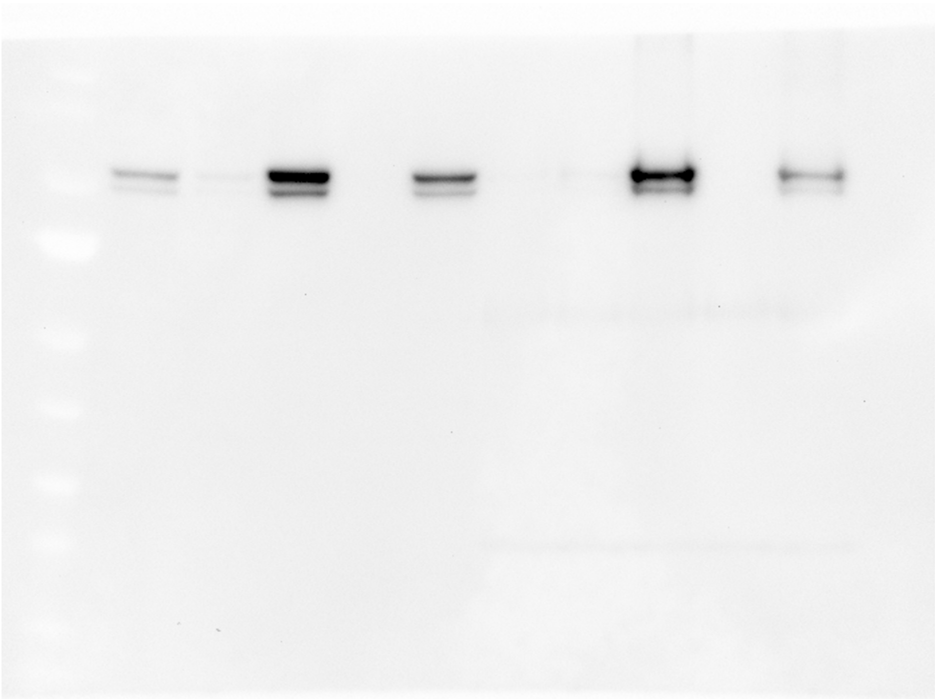

Figure 7 Source Data 2. Mouse Dux co-IP, anti-pSTAT1(Y701). Western blot showing anti-pSTAT1(Y701) signal for Figure 7b. * marks correct size bands. Blot was stripped from previous exposure and re-probed with anti-pSTAT1(Y701). Protein ladder only appears in the “white light” exposure, signal from ECL only appears in the chemiluminescence channel. The double-bands marked by the * represent the alpha (upper) and beta (lower) isoforms of endogenous STAT1.
