# Supplementary material for: Human DUX4 and mouse Dux interact with STAT1 and broadly inhibit interferon-stimulated gene induction
Source: eLife. 2023 Apr 24;12:e82057. doi: 10.7554/eLife.82057 (PMC10195082; doi:10.7554/eLife.82057)
Supplement: Figure 7—source data 3. — Western blot showing anti-pSTAT1(S727) signal for Figure 7B. * marks correct size band. Blot was stripped from previous exposure and re-probed with anti-pSTAT1(S727). Protein ladder only appears in the ‘white light’ exposure. Signal from ECL only appears in the chemiluminescence channel. Only STAT1-alpha can be phosphorylated at S727, hence the lack of double band. [file elife-82057-fig7-data3.zip › Figure7-SourceData3.pdf]

white light:

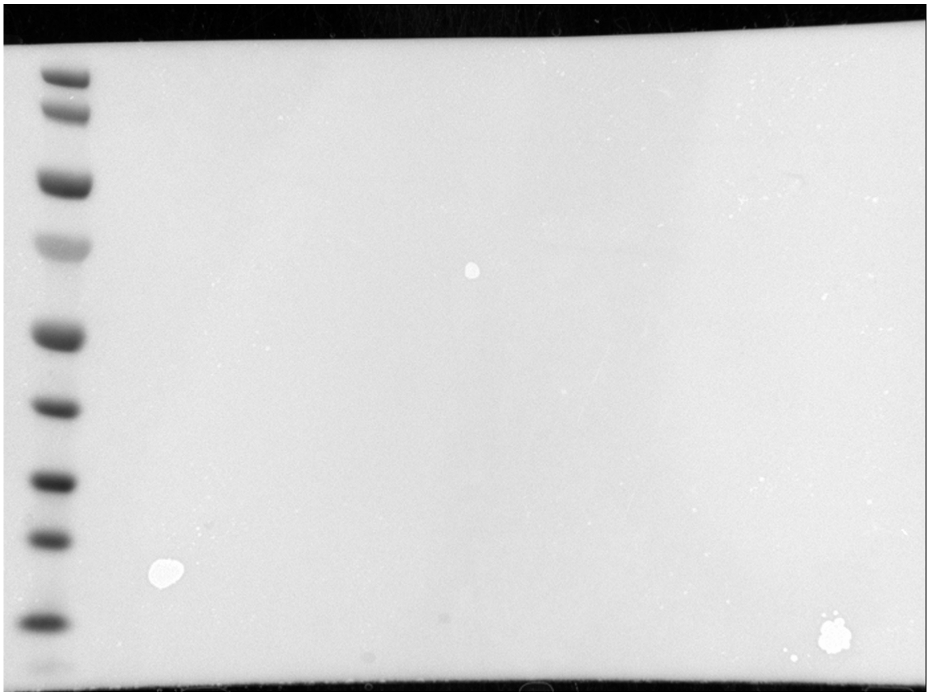

*

chemiluminescence:

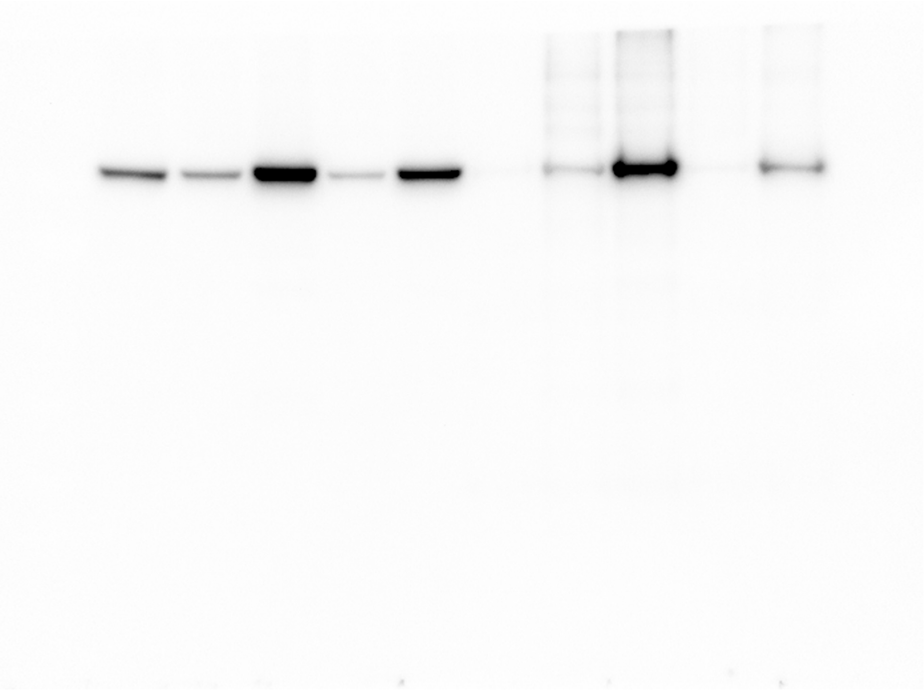

Figure 7 Source Data 3. Mouse Dux co-IP, anti-pSTAT1(S727). Western blot showing anti-pSTAT1(S727) signal for Figure 7b. * marks correct size band. Blot was stripped from previous exposure and re-probed with anti-pSTAT1(S727). Protein ladder only appears in the “white light” exposure, signal from ECL only appears in the chemiluminescence channel. Only STAT1-alpha can be phosphorylated at S727, hence the lack of double-band.
