# Supplementary material for: Human DUX4 and mouse Dux interact with STAT1 and broadly inhibit interferon-stimulated gene induction
Source: eLife. 2023 Apr 24;12:e82057. doi: 10.7554/eLife.82057 (PMC10195082; doi:10.7554/eLife.82057)
Supplement: Figure 7—source data 4. — Western blot showing anti-FLAG signal for Figure 7B. * marks correct size band. Blot was physically cut to probe with multiple antibodies, multiple unrelated blots were imaged in this exposure/file. Lower blot (boxed in green) is probed with anti-FLAG to detect inducible FLAG-tagged transgenes. Note that the image has been flipped in the article and labeled appropriately. Protein ladder only appears in the ‘white light’ exposure. Signal from ECL only appears in the chemiluminescence channel. [file elife-82057-fig7-data4.zip › Figure7-SourceData4.pdf]

white light:

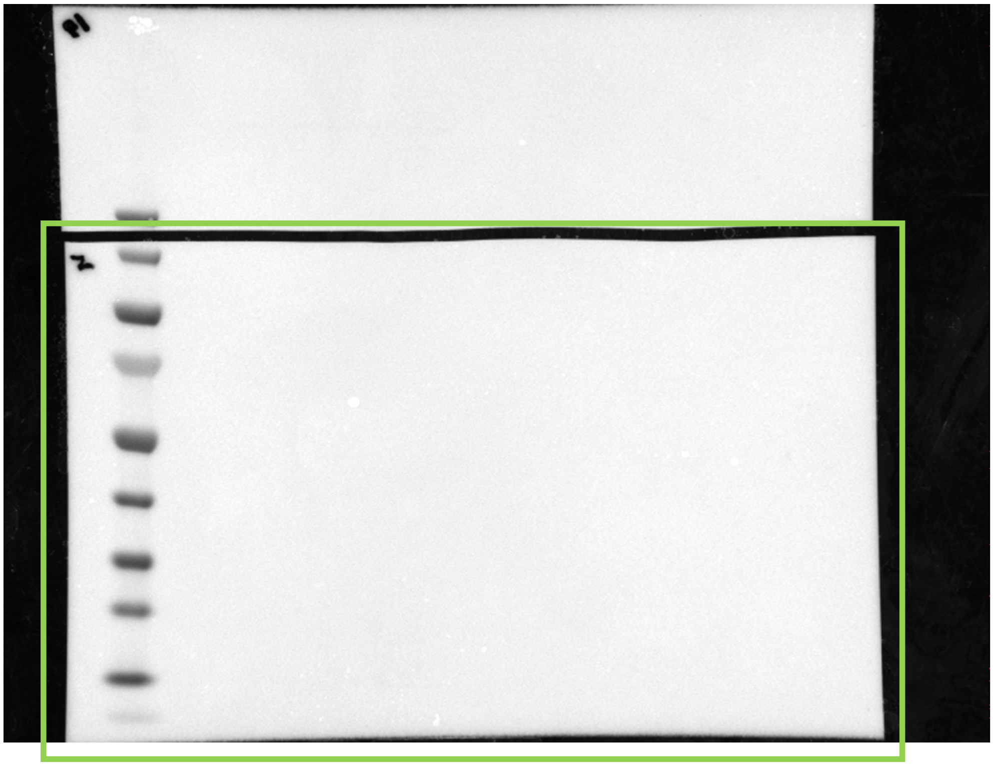

chemiluminescence:

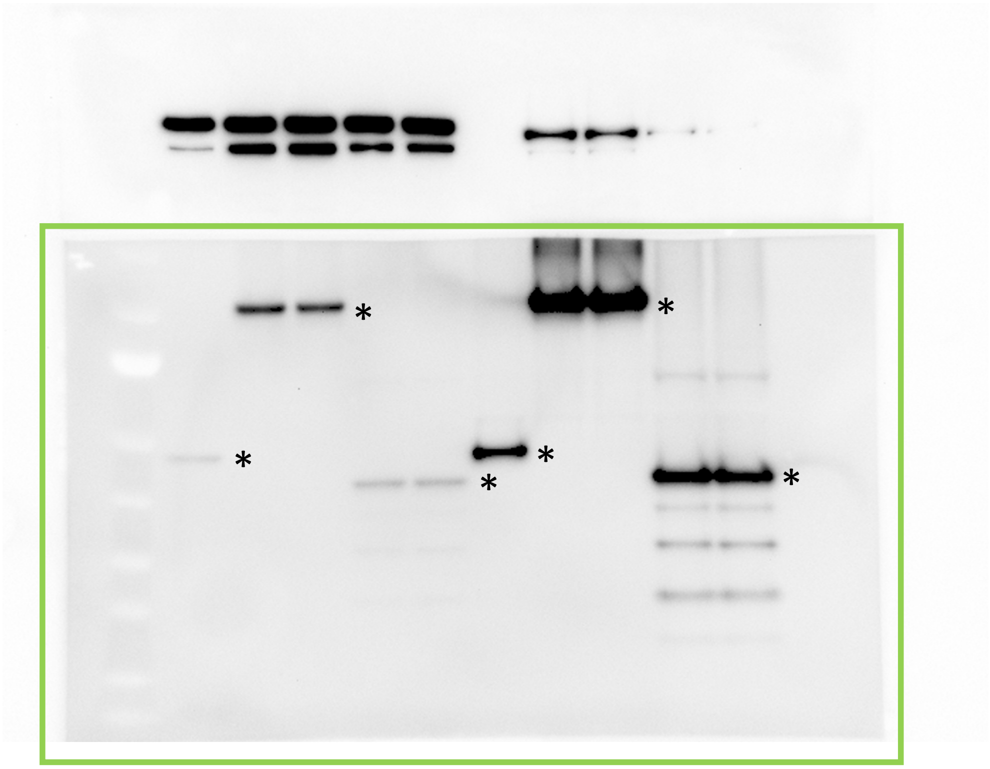

Figure 7 Source Data 4. Mouse Dux co-IP, anti-FLAG. Western blot showing anti-FLAG signal for Figure 7b. * marks correct size band. Blot was physically cut to probe with multiple antibodies, multiple unrelated blots were imaged in this exposure/file. LOWER BLOT (boxed in green) is probed with anti-FLAG to detect inducible FLAG-tagged transgenes. NOTE: The image has been flipped in the manuscript and labeled appropriately. Protein ladder only appears in the "white light" exposure, signal from ECL only appears in the chemiluminescence channel.
